# *Mycobacterium paratuberculosis* zoonosis is a One Health emergency

**DOI:** 10.1007/s10393-022-01602-x

**Published:** 2022-06-02

**Authors:** Coad Thomas Dow, Briana Lizet Alvarez

**Affiliations:** 1 Department of Ophthalmology and Visual Sciences, 9431 Wisconsin Institutes for Medical Research (WIMR), McPherson Eye Research Institute, University of Wisconsin-Madison, 1111 Highland Avenue, Madison, WI 53705 USA; 2 Biology and Global Health, University of Wisconsin-Madison, 120 N Orchard St #1, Madison, WI 53705 USA

**Keywords:** Mycobacterium avium ss. paratuberculosis, zoonosis, Johne's disease, Crohn's disease, autoimmune diabetes, One Health

## Abstract

A singular pathogen has been killing animals, contaminating food and causing an array of human diseases. *Mycobacterium avium* subspecies *paratuberculosis* (MAP) is the cause of a fatal enteric infectious disease called Johne’s (Yo’-nees), a disorder mostly studied in ruminant animals. MAP is globally impacting animal health and imparting significant economic burden to animal agriculture. Confounding the management of Johne’s disease is that animals are typically infected as calves and while commonly not manifesting clinical disease for years, they shed MAP in their milk and feces in the interval. This has resulted in a “don’t test, don’t tell” scenario for the industry resulting in greater prevalence of Johne’s disease; furthermore, because MAP survives pasteurization, the contaminated food supply provides a source of exposure to humans. Indeed, greater than 90% of dairy herds in the US have MAP-infected animals within the herd. The same bacterium, MAP, is the putative cause of Crohn’s disease in humans. Countries historically isolated from importing/exporting ruminant animals and free of Johne’s disease subsequently acquired the disease as a consequence of opening trade with what proved to be infected animals. Crohn’s disease in those populations became a lagging indicator of MAP infection. Moreover, MAP is associated with an increasingly long list of human diseases. Despite MAP scientists entreating regulatory agencies to designate MAP a “zoonotic agent,” it has not been forthcoming. One Health is a global endeavor applying an integrative health initiative that includes the environment, animals and humans; One Health asserts that stressors affecting one affects all three. Recognizing the impact MAP has on animal and human health as well as on the environment, it is time for One Health, as well as other global regulatory agencies, to recognize that MAP is causing an insidious slow-motion tsunami of zoonosis and implement public health mitigation.

## Introduction

As global populations begin to recover from the recent pandemic caused by coronavirus disease 2019 (COVID-19), attention has turned to the origin of the disease found at the interface between animals and humans. Where did this disease originate? Molecular and serologic evidence points to bats (Zhou et al. 2020) and pangolins (Xiao et al. 2020), both species having previously been associated with SARS-CoV-2-related viruses (Wacharapluesadee et al. 2021). A global movement that addresses health risk, including zoonosis, occurring at the human–animal–environment interface is called One Health (Vandersmissen and Welburn 2014).

This integrated approach to health across disciplines received formal recognition with the establishment in 2010 of collaborative agreement between the World Health Organization (WHO), World Organization for Animal Health (OIE) and the Food and Agriculture Organization (FAO) to “address health risks at the animal–human–ecosystems interface” (FAO–OIE–WHO 2021).

While some zoonotic diseases such as anthrax, bovine tuberculosis and brucellosis attract significant attention from the international health community, less common zoonotic diseases are considered ‘neglected’ and are inadequately addressed nationally and internationally. The World Health Organization designates these Neglected Zoonotic Diseases (NZD).

A common, yet often neglected, zoonotic pathogen is *Mycobacterium avium* subspecies *paratuberculosis* (MAP) (The editors 2002). MAP is the well-established cause of a fatal enteritis mostly studied in ruminant animals called Johne’s (Yo’-nees) disease (Johne’s Information Center 2021).

## Johne’s Disease—Paratuberculosis of Animals

Johne's disease of ruminant animals is a common, contagious, chronic, granulomatous enteritis characterized by persistent and progressive diarrhea, weight loss and death (Whittington and Sergeant 2001). It is costly to dairy farming as it causes reduced milk production, increased mortality and premature culling of sick animals as well as reduced sale price for cattle from regions with a high disease burden (Marcé et al. 2010). Johne’s disease is primarily transmitted by the fecal–oral route with MAP exposure during consumption of milk or colostrum containing MAP bacilli. Animal exposure to MAP can also come from contaminated pastures, feed, soil and/or water (Whittington and Sergeant 2001; Stabel 2006).

Newborn ruminants are more susceptible than adults presumably due to their relatively undeveloped immune system (Stabel 2006). Yet adult cattle, having exposure to high MAP inocula, can acquire both infection and disease (Stewart et al. 2004; Roermund et al. 2007; Whittington et al. 2012). After infection, disease progression follows four distinct stages: latent, subclinical, clinical and advanced (Whittington et al. 2012; Whitlock and Buergelt 1996). Infected cattle begin shedding bacilli after a latent period ranging from 2 to 10 years; shedding of MAP increases with disease progression.

When an infected animal is identified, it reflects herd transmission events that occurred years earlier. The finding of a clinically infected animal is the “tip of the iceberg,” alluding to the high background prevalence of undiagnosed, subclinical infection of animas. For example, it is estimated that for each animal in the advanced stage, there are 1-2 animals in the clinical stage, 4-8 in the subclinical stage and 10-14 in the latent stage (Magombedze et al. 2013). According to the United States Department of Agriculture (USDA), herd-level prevalence of MAP infection in US dairy herds increased from 21.6% in 1996 to 91.1% in 2007 (Lombard et al. 2007).

India extensively tested ruminant animals for MAP burden and reported an increasing MAP “bio-load” in cattle (43%), buffalo (36%), goats (23%) and sheep (41%). Moreover, in this same geographic area, 30.8% of 28,291 humans (via serum ELISA, blood PCR and stool PCR) tested positive for MAP (Chaubey et al. 2017). Similarly, testing of ruminants in Saudi Arabia found MAP; 26% of sheep, 27% of goat, 30% of cattle and 15% of camels (Elsohaby et al. 2021).

### MAP in the Environment

MAP is a resilient organism and shedding by infected animals is a major source of environmental MAP; once excreted, MAP can survive up to 120 weeks in soil or water (Garvey 2020). MAP is found in grazing areas as well as in runoff continuing on to rivers and in municipal water (Beumer et al. 2010). These water sources may be a significant reservoir of MAP (Whittington et al. 2005) as it persists in the biofilm (Botsaris et al. 2016). Cow manure in solid and liquid forms is applied as fertilizer to agricultural land (Grewal et al. 2006). MAP persists on farms depopulated of ruminants. MAP persists in the soil and grass of pasture plots (Whittington et al. 2004); persisting in both the root and aerial parts of plants (Kaevska et al. 2014; Rhodes et al. 2014). Aerosol inhalation is another suggested route of transmission of MAP to animals (Corner et al. 2004) and possibly humans as MAP is found in river aerosols as well as domestic showers (Rhodes et al. 2014; Pickup et al. 2005; Eisenberg et al. 2011).

The inefficiency of MAP diagnostic tests coupled with the long latency of infection in seemingly uninfected, productive animals makes producers hesitant to test their herds—and there is no mandate to do so. This results in (1) MAP-shedding animals that contaminate products (2) trade of asymptomatic animals and 3) delayed animal culling (Garvey 2020 Oct 1).

### MAP in Food

Milk and dairy products are considered to be the primary source of MAP infection in humans (Gill et al. 2011); products from pasteurized milk constitute a consumption risk as pasteurization only reduces the MAP load originally present in milk (Gill et al. 2011; Eltholth et al. 2009). MAP is present in yogurt (Brandt et al. 2011), cheese (Galiero et al. 2016), muscle meat (Alonso-Hearn et al. 2009) and hamburger (Hammer et al. 2013).

### MAP and Human Disease

Though the link of MAP zoonosis to Crohn’s disease has been controversial for over one hundred years (Sechi and Dow 2015), validation of this association has come from studies showing Crohn’s disease resolution with anti-mycobacterial therapy targeted against MAP (Qasem et al. 2020; Agrawal et al. 2020; Savarino et al. 2019; Borody et al. 2007). Moreover, MAP is now linked to an increasing list of inflammatory and autoimmune diseases (Dow and Sechi 2019; Ekundayo and Okoh 2020). To date, MAP has been causally associated with granulomatous diseases: Crohn’s (Kuenstner et al. 2017), sarcoidosis (Celler BG 2018; Reid and Chiodini 1993) and Blau syndrome (Dow and Ellingson 2010). Through molecular mimicry from mycobacterial heat shock protein (hsp65) (Dow 2012), MAP induces autoantibodies in autoimmune diabetes (T1D) (Naser et al. 2013 Jun), multiple sclerosis (Cossu et al. 2013; Ekundayo et al. 2022), autoimmune thyroiditis (Sisto et al. 2010), lupus (Dow 2016), rheumatoid arthritis (Bo et al. 2019a, 2018) and possibly, Sjogren’s syndrome (Dow and Chan 2021). The causal association of three diseases will be further featured: T1D, multiple sclerosis and rheumatoid arthritis.

### MAP and Type 1 Diabetes

Type 1 diabetes (T1D) is a chronic autoimmune disease (Eisenbarth 1986); it is associated with early life dietary exposure to cow’s milk. A large international study of 78 centers in 15 countries was conducted; the TRIGR (Trial to Reduce Insulin-Dependent Diabetes Mellitus in the Genetically at Risk) Study was initiated with the rationale that cows’ milk protein is too complex and that early exposure to it will provoke an autoimmune response in at-risk infants. Two study arms used cows’ milk-based formula; one arm had traditionally prepared formula while the other had extensively hydrolyzed formula. The results: it did not work; “Weaning to a hydrolyzed formula did not reduce the risk of type 1 diabetes in children with an increased disease risk” (Knip et al. 2018).

An alternative explanation encompassing the rationale for the study was presented in 2018 (Dow 2018). It proposed that *Mycobacterium avium* ss. *paratuberculosis* (MAP), present in the formula, was the trigger for autoimmune diabetes. It suggested that shared genetic risk for both mycobacterial infection and T1D offers a permissive environ for latent MAP infection in the infant. Further, MAP’s immunodominant heat shock protein 65 (HSP65) cross reacts with pancreatic glutamic acid decarboxylase (GAD) through molecular mimicry (Vandersmissen and Welburn 2014) resulting in anti-GAD antibodies causing an immune mediated destruction of insulin producing islet cells of the pancreas.

In 2006, Dow postulated that MAP may be an environmental trigger for T1D in the genetically at-risk. Three proposals were offered to support the postulate: (1) there are shared genetic susceptibilities to both mycobacterial infection and T1D, (2) MAP is the source of the HSP65 protein, providing epitope homologies between mycobacterial HSP65 and pancreatic glutamic acid decarboxylase (GAD) and (3) epidemiologic findings tie the risk of T1D to early life exposure to cow’s milk (Dow 2006). Subsequently, Sechi and associates conducted several studies associating MAP and T1D. They found an association of MAP and T1D patients on their home island of Sardinia (Sechi et al. 2008; Sechi et al. 2008; Cossu et al. 2011). The island of Sardinia has the second highest incidence of T1D in the world (Songini et al. 2017). They reported finding MAP in T1D patients but not in type 2 diabetics (Rosu et al. 2008; Rosu et al. 2009). They found MAP in T1D children (Bitti et al. 2012; Cossu et al. 2013; Masala et al. 2013). They confirmed a genetic risk factor linking mycobacterial infection and T1D (Paccagnini et al. 2009). They also identified additional MAP peptides that are homologous with pancreatic proteins (Cossu et al. 2011; Masala et al. 2011; Scotto et al. 2012) and showed that immune reaction to these MAP peptides cross-react to the classical islet cell antibodies (Niegowska et al. 2016). They demonstrated parallel findings on the Italian mainland (Masala et al. 2014; Masala et al. 2015) and (Sechi and collaborators) in Iran (Hesam Shariati et al. 2016).

Recently, a body of evidence pointed to a role for human endogenous retroviruses (HERVs) in the activation of genes (Greenig 2019). It is thought that most HERVs are genetically silent. However, assorted environmental stimuli, including infection, may activate HERVs to potentiate certain autoimmune diseases (Levet et al. 2017). A recent study demonstrated anti-HERV antibodies correlating with sero-reactivity against MAP in children at risk for T1D (Niegowska et al. 2019). This study showed that an activated HERV gene expressing a specific envelope protein, HERV-W, is associated with T1D in diverse populations.

Of more than a dozen articles implicating MAP in T1D, only one article failed to do so. That article came from India where MAP was not found in the blood of T1D patients. A few possible explanations offered included the compulsory BCG vaccination against tuberculosis, with the thought that BCG provides cross protection against paratuberculosis as it does with leprosy. Also, the cultural culinary practice of vegetarianism would reduce exposure to MAP, as would the common practice of boiling milk before consumption (Rani et al. 2014).

Of interest is the publication that the BCG vaccination of long standing T1D individuals, followed by a booster in one month, resulted in the control of blood sugar (seen after a delay of three years). The effect was durable with normal blood sugars eight years after the vaccination (Kühtreiber and Faustman 2019). The beneficial effect is postulated to be due to a “reset” of the immune system. An alternative explanation is that BCG vaccination is effective against MAP as it is against tuberculosis and non-tuberculous mycobacteria (Dow 2018).

### MAP and Multiple Sclerosis

There has been steady progress in the identification of microbial triggers of multiple sclerosis; this includes animal model studies of experimental autoimmune encephalomyelitis (EAE) a surrogate model of multiple sclerosis. The introduction of MAP both orally and subcutaneously has been shown to elicit EAE (Cossu et al. 2021, 2019).

Several studies have demonstrated the existence of a link between MAP and multiple sclerosis in Italy. Similar testing was carried out in Japan. The findings support the view that MAP acts as a risk factor or a triggering agent of multiple sclerosis in some Japanese patients with genetic susceptibility to the mycobacterium (Cossu et al. 2016). The proliferation of Italian MAP-autoimmune studies came primarily from the specialized lab of Prof. Leonardo Sechi, Sardinia. His postdoctoral student, Davide Cossu, matriculated to the lab of Prof. Eiichi Momotani in Japan. Cossu continued his investigation of microbial triggers of multiple sclerosis there and coauthored several publications that have linked MAP as well as other microbial triggers of multiple sclerosis in these two disparate populations (Ekundayo et al. 2022; Cossu et al. 2017).

Antigenic peptides of both MAP and Epstein-Barr virus (EBV) are recognized by anti-myelin basic protein in multiple sclerosis individuals supporting the concept that both MAP and EBV trigger multiple sclerosis autoimmunity through a common target (Mameli et al. 2014).

Anti-MAP antibodies are identified in the spinal fluid of, not only multiple sclerosis individuals, but also those with the clinically distinct, but related disease, neuromyelitis optica spectrum disorder (Yokoyama et al. 2018).

A marked media response was seen to the January 2022 issue of the journal *Science* wherein a report from a large database revealed a high prevalence of the EBV in association with multiple sclerosis (Bjornevik et al. 2022). While the scope of the data mining was large, this revelation was not novel; paralleling the previous section on RA, identified multiple sclerosis-related microbial antigens come from EBV, MAP and HERVs (Frau et al. 2021).

### MAP and Rheumatoid Arthritis

The uptake and survival of MAP in human cells is enhanced in cholesterol-rich compartments that are slow to acidify (Keown et al. 2012). MAP, like other pathogenic mycobacteria, is able to manipulate host lipid metabolism and accumulate cholesterol within macrophages to enhance infection (Johansen et al. 2019 May). This association between host lipoprotein levels and reactivity to MAP is seen in autoimmune diabetes, rheumatoid arthritis (RA) and multiple sclerosis (Bo et al. 2019b).

Many studies have tied MAP to RA. MAP virulence factors tyrosine phosphatase A (PtpA) and kinase G (PknG) are proteins necessary for MAP survival within macrophages. PtpA and PknG are strongly recognized in RA which supports the hypothesis that MAP infection may be involved in the pathogenesis of RA (Bo et al. 2019a). Moreover, polymorphisms of tumor necrosis factor (TNF) receptors which are linked to Crohn’s disease are associated with RA as well as poor response in some patients to ant-TNF treatment (Naser et al. 2019).

Specific genetic polymorphisms that regulate immune responses that increase susceptibility to mycobacterial infection and Crohn’s disease (Sharp et al. 2018) are also found in RA. The *Protein Tyrosine Phosphatase Non-receptor type 2 and 22* (*PTPN2/22)* polymorphisms found in RA patients who also had the DNA of MAP in their blood (Sharp et al. 2018). RA is characterized by erosive joint damage as well as by cellular and humoral responses against a broad range of self-peptides. Interferon regulatory factor 5 (IRF5) is such an RA peptide and a mimicry target of both Epstein-Barr virus (EBV) antigen BOLF1 and MAP antigen MAP_4027; this supports the hypothesis that both EBV and MAP infections may be involved with the pathogenesis of RA by triggering an immune response against RA self-peptides (Bo et al. 2018).

A recent study suggests a role for multiple microbial antigens in the etiology of RA: MAP, EBV, and the human endogenous retrovirus (HERV); all were shown to exhibit a humoral immune response in RA individuals compared to controls (Jasemi et al. 2021). HERVs are ancient viruses that have been integrated into the human genome. Most often silent, they have been associated with several autoimmune diseases (Balada et al. 2010).

### MAP Zoonosis Principles—Parsimony and Precautionary

*Although MAP* is difficult to detect and even more difficult to culture, it is significantly associated with Crohn’s disease and, if appropriate culture and PCR tests are done correctly, nearly every individual with chronic inflammation of the gut from Crohn’s disease is found to be infected with MAP (Feller et al. 2007; Bull et al. 2003; Naser et al. 2004; Sabatino et al. 2011).

## Parsimony

Occam’s razor, or the principle of parsimony, could be employed in the MAP / Crohn’s zoonosis debate. The principle roughly states: the simplest explanation is usually the right one. Some have argued that the causation of Crohn’s by MAP is already solidified citing fulfilled Koch’s postulates as well as Relman criteria (Chamberlin et al. 2007; Agrawal et al. 2014).

In 1930, paratuberculosis was unknown in Iceland when sheep were imported from Germany and distributed to fourteen farms (Fridriksdottir et al. 2000). The apparently healthy, yet sub-clinically infected animals brought paratuberculosis to Iceland and by 1938 five of the original farms had infected sheep. By 1945, clinical paratuberculosis was found in cattle on the same farms and the organism was later confirmed as the sheep strain of MAP by molecular techniques (Whittington et al. 2001). The incidence of Crohn’s disease is noted to have steadily increased by 14-fold over the last half of the twentieth century (Hruska and Pavlik 2014).

Similarly, before 1990 paratuberculosis was virtually unknown in Czechoslovakia; a country isolated economically and politically until 1989 when the Iron Curtain was lifted. Following that came opened borders and importation of livestock. Now, as the Czech Republic and possessing comprehensive medical records, an increase in Crohn’s disease of more than 13-fold occurred between 1995 and 2012 (Hruska and Pavlik 2014).

A comprehensive review of pediatric inflammatory bowel disease in thirty-eight countries between 1985 and 2018 found a steadily increasing incidence of Crohn’s disease and concluded that the results indicate its emergence as a global disease; moreover, the authors suggest that studies should investigate environmental risk factors for pediatric cohorts (Sýkora et al. 2018).

### Precautionary Principle

The precautionary principle is a policy making approach that considers adoption of preventative measures to address potential risks to the environment and/or to the public. Transmissible Animal Diseases and Food Safety (TASF) is a Swiss-based international forum. TASF acknowledges the uncertainties of the zoonotic potential of MAP and suggests:

“… a decision by food safety regulators to exercise the ‘precautionary principle’, label MAP as a potential zoonotic agent, and adopt measures to limit as much as possible the levels of MAP contamination of raw milk and meat would go far to protect the coming generations of children from MAP exposure, possible infection, and potentially Crohn’s disease.” (Transmissible Animal Diseases and Food Safety Forum 2022).

Similarly, the United Kingdom has produced a general statement referencing the United Kingdom Food Standards Agency policy towards MAP and human health, advising that the precautionary principle be adopted (Agri-Food & biosciences Institute 2022).

A study conducted among Australian veterinarians regarding their perceptions of the MAP /Crohn’s causation debate revealed that nearly one third viewed MAP as the likely cause of Crohn’s disease and the other two thirds agreed with the adoption of the precautionary principle (Acharya et al. 2020). The precautionary principal discussions have been entrenched around MAP zoonosis and Crohn’s disease. Another inflammatory disease associated with MAP is sarcoidosis (Reid and Chiodini 1993); the first case report of sarcoidosis cured with anti-MAP antibiotics has been reported (Celler 2018).

## Discussion—MAP zoonosis: Parsimony Insight and Precaution Incite

**Figure 1:**
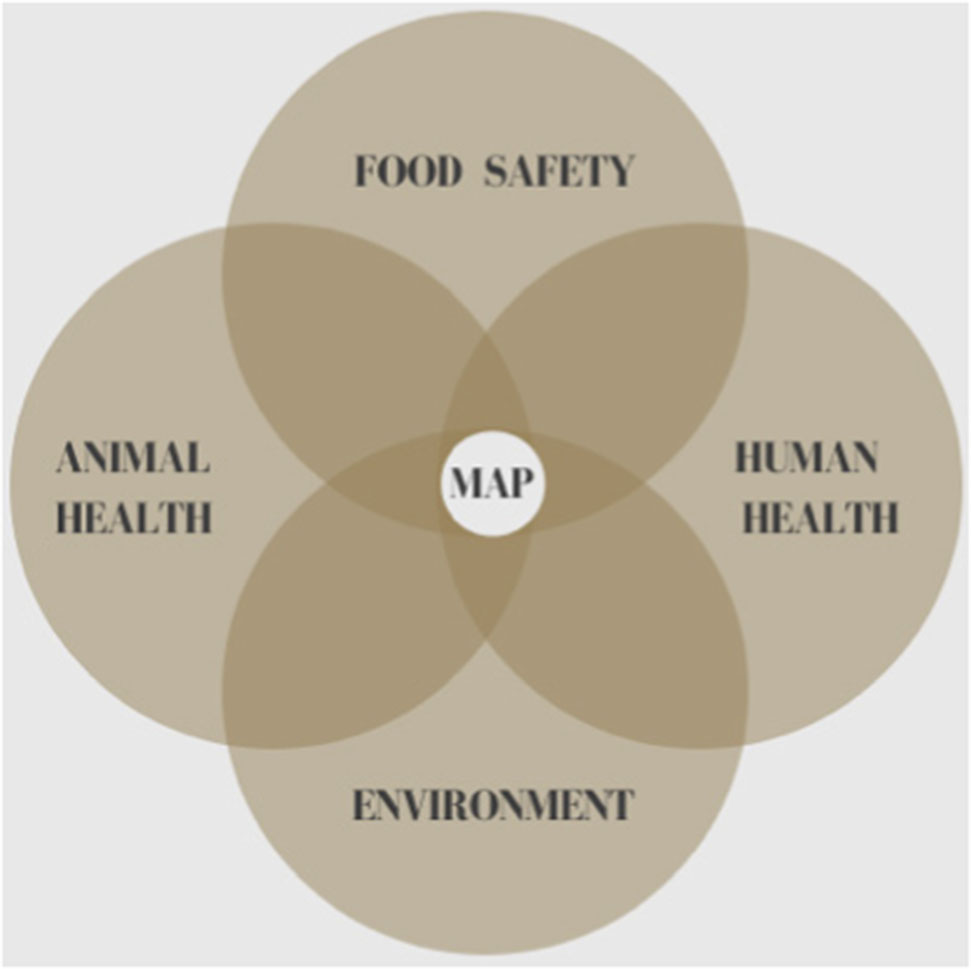
*Mycobacterium avium* subspecies *paratuberculosis*—MAP—is the cause of Johne’s disease of ruminant animals. MAP contaminates food, the environment and is associated with an increasing list of human inflammatory and autoimmune diseases. One Health is uniquely positioned to introduce and advance policies that address the consequences of MAP in the environment and food as well as in animal and human health.

The incidence of T1D in children is increasing worldwide (Hummel et al. 2012) as is the incidence of Crohn’s disease (Torres et al. 2017). Both the principle of parsimony and Koch’s postulates support inculpation of MAP as a cause of Crohn’s disease. Regardless of the relative strength one might assign to the MAP/Crohn’s association, this article enumerates other MAP-associated diseases and the increasing medical literature supporting it (Ekundayo and Okoh 2020). The combined weight of these disease associations should incite a call to action by regulatory agencies to invoke the precautionary principle with regard to consumption of MAP-contaminated food in at-risk individuals. In spite of public health implications, contamination of milk and dairy products with MAP is not currently restricted. We view the management of public health risk due to MAP as an increasingly important policy issue. With mounting global recognition of the impact MAP has upon the health of the environment, animals and humans, One Health is well positioned at that nexus (Fig. 1). One Health is in a unique position to elevate the discussion to mitigate this emerged, yet neglected zoonotic pathogen: *Mycobacterium avium* subspecies *paratuberculosis*.
